# Supplementary material for: Morbidity and unplanned healthcare encounters after hospital discharge among young children in Dar es Salaam, Tanzania and Monrovia, Liberia
Source: BMJ Paediatr Open. 2024 Jun 21;8(1):e002613. doi: 10.1136/bmjpo-2024-002613 (PMC11191828; doi:10.1136/bmjpo-2024-002613)
Supplement: Supplementary data [file bmjpo-2024-002613supp001.pdf]

Supplemental Table 1. Comparison of timing of symptoms reported among participants who died and those who survived up to 60 days following hospital discharge

Type of Healthcare Sought	Survived 60 Days After Discharge, n (%)	Died within 60 Days After Discharge, n (%)	*P* value
Day 7			
None	2,834 (76%)	72 (56%)	<0.001
Common cold	136 (3.6%)	2 (1.6%)	0.45
Cough	385 (10%)	15 (13%)	0.43
Difficulty breathing	24 (0.6%)	14 (12%)	<0.001
Fever	436 (12%)	13 (11%)	0.79
Refusal to eat, drink, or breastfeed	319 (8.5%)	25 (21%)	<0.001
Pus draining from ear	4 (0.1%)	1 (0.8%)	0.15
Vomiting	36 (1.0%)	3 (2.5%)	0.12
Abdominal pain	5 (0.1%)	1 (0.8%)	0.18
Anemia	3 (<0.1%)	0 (0%)	>0.99
Diarrhea	8 (0.2%)	0 (0%)	>0.99
Fussiness	2 (<0.1%)	2 (1.6%)	0.01
Injury	0 (0%)	0 (0%)	--
Jaundice	11 (0.3%)	3 (2.3%)	0.01
Rash	5 (0.1%)	1 (0.8%)	0.18
Seizure	5 (0.1%)	1 (0.8%)	0.18
Weight loss	0 (0%)	0 (0%)	--
Other	2 (<0.1%)	3 (2.5%)	<0.001
Day 14			
None	3,139 (83%)	60 (55%)	<0.001
Common cold	100 (2.6%)	4 (3.7%)	0.36
Cough	311 (8.2%)	20 (19%)	<0.001
Difficulty breathing	16 (0.4%)	10 (9.6%)	<0.001
Fever	333 (8.8%)	20 (19%)	<0.001
Refusal to eat, drink, or breastfeed	133 (3.5%)	14 (13%)	<0.001
Pus draining from ear	1 (<0.1%)	0 (0%)	>0.99
Vomiting	11 (0.3%)	3 (2.9%)	0.005
Abdominal pain	3 (<0.1%)	2 (1.8%)	0.01
Anemia	0 (0%)	0 (0%)	--
Diarrhea	3 (<0.1%)	2 (1.8%)	0.01
Fussiness	1 (<0.1%)	1 (0.9%)	0.06
Injury	3 (<0.1%)	0 (0%)	>0.99
Jaundice	8 (0.2%)	0 (0%)	>0.99
Rash	3 (<0.1%)	0 (0%)	>0.99
Seizure	4 (0.1%)	1 (0.9%)	0.13
Weight loss	1 (<0.1%)	0 (0%)	>0.99
Other	6 (0.2%)	2 (1.9%)	0.02
Day 30			
None	2,766 (73%)	47 (56%)	<0.001
Common cold	163 (4.3%)	2 (2.4%)	0.77
Cough	471 (12%)	9 (12%)	0.90
Difficulty breathing	23 (0.6%)	13 (17%)	<0.001
Fever	454 (12%)	18 (24%)	0.002
Refusal to eat, drink, or breastfeed	246 (6.5%)	6 (8.0%)	0.63
Pus draining from ear	5 (0.1%)	0 (0%)	>0.99
Vomiting	25 (0.7%)	1 (1.3%)	0.40
Abdominal pain	7 (0.2%)	4 (4.8%)	<0.001
Anemia	2 (<0.1%)	1 (1.2%)	0.06
Diarrhea	10 (0.3%)	0 (0%)	>0.99
Fussiness	2 (<0.1%)	3 (3.6%)	<0.001
Injury	0 (0%)	0 (0%)	--
Jaundice	2 (<0.1%)	1 (1.2%)	0.06
Rash	9 (0.2%)	0 (0%)	>0.99
Seizure	3 (<0.1%)	2 (2.4%)	0.004

Kisenge R, *et al. BMJ Paediatrics Open* 2024; 8:e002613. doi: 10.1136/bmjpo-2024-002613

Weight loss	0 (0%)	1 (1.2%)	0.02
Other	10 (0.3%)	1 (1.3%)	0.19
Day 45			
None	3,008 (83%)	27 (47%)	<0.001
Common cold	105 (2.9%)	5 (8.8%)	0.01
Cough	264 (7.3%)	6 (13%)	0.15
Difficulty breathing	14 (0.4%)	13 (28%)	<0.001
Fever	259 (7.1%)	10 (22%)	0.001
Refusal to eat, drink, or breastfeed	161 (4.4%)	10 (22%)	<0.001
Pus draining from ear	5 (0.1%)	0 (0%)	>0.99
Vomiting	15 (0.4%)	2 (4.3%)	0.02
Abdominal pain	7 (0.2%)	1 (1.8%)	0.12
Anemia	3 (<0.1%)	0 (0%)	>0.99
Diarrhea	3 (<0.1%)	2 (3.5%)	0.002
Fussiness	1 (<0.1%)	0 (0%)	>0.99
Injury	1 (<0.1%)	0 (0%)	>0.99
Jaundice	3 (<0.1%)	1 (1.8%)	0.06
Rash	6 (0.2%)	0 (0%)	>0.99
Seizure	3 (<0.1%)	0 (0%)	>0.99
Weight loss	0 (0%)	1 (1.8%)	0.02
Other	11 (0.3%)	4 (8.7%)	<0.001
Day 60			
None	3,748 (90%)	14 (37%)	<0.001
Common cold	94 (2.2%)	2 (5.3%)	0.16
Cough	177 (4.2%)	2 (6.3%)	0.40
Difficulty breathing	18 (0.4%)	12 (38%)	<0.001
Fever	184 (4.4%)	6 (19%)	0.003
Refusal to eat, drink, or breastfeed	88 (2.1%)	6 (19%)	<0.001
Pus draining from ear	11 (0.3%)	0 (0%)	>0.99
Vomiting	12 (0.3%)	2 (6.3%)	0.005
Abdominal pain	3 (<0.1%)	1 (2.6%)	0.04
Anemia	1 (<0.1%)	0 (0%)	>0.99
Diarrhea	12 (0.3%)	1 (2.6%)	0.11
Fussiness	1 (<0.1%)	1 (2.6%)	0.02
Injury	2 (<0.1%)	0 (0%)	>0.99
Jaundice	0 (0%)	2 (5.3%)	<0.001
Rash	6 (0.1%)	0 (0%)	>0.99
Seizure	3 (<0.1%)	2 (5.3%)	<0.001
Weight loss	1 (<0.1%)	1 (2.6%)	0.02
Other	9 (0.2%)	2 (6.3%)	0.003

Kisenge R, *et al. BMJ Paediatrics Open* 2024; 8:e002613. doi: 10.1136/bmjpo-2024-002613

Supplemental Table 2. Median proportion of symptomatic days after hospital discharge among children who survived 60 days and those who died within 60 days of hospital discharge*

Symptoms Reported by Caregiver	Median Proportion (%) of Days with Symptoms among those who Survived 60 Days After Discharge, median (IQR)	Median Proportion (%) of Days with Symptoms among those who Died within 60 Days After Discharge, median (IQR)	*P* value
All Participants			
Common cold	3.33 (1.67, 5.00)	2.78 (2.22, 12.50)	0.28
Cough	5.00 (3.30, 6.70)	8.90 (5.00, 16.70)	<0.001
Difficulty breathing	3.00 (2.00, 5.00)	3.00 (2.00, 14.00)	0.01
Fever	5.00 (3.30, 6.70)	7.50 (4.20, 21.90)	<0.001
Refusal to eat, drink, or breastfeed	5.00 (3.30, 6.70)	7.10 (2.20, 21.40)	<0.001
Pus draining from ear	1.67 (1.67, 3.33)	14.29 (14.29, 14.29)	0.07
Vomiting	3.30 (1.70, 5.00)	3.30 (1.80, 11.80)	0.68
Abdominal pain	3.00 (2.00, 5.00)	20.00 (11.00, 36.00)	0.01
Anemia	5.00 (1.67, 6.67)	10.00 (10.00, 10.00)	0.16
Diarrhea	3.33 (1.67, 4.17)	8.25 (2.08, 15.16)	0.32
Fussiness	3.00 (2.00, 4.00)	3.00 (3.00, 11.00)	0.21
Injury	3.33 (1.67, 3.33)	-- (--, --)	--
Jaundice	5.00 (3.30, 8.30)	15.20 (7.50, 33.90)	0.12
Rash	5.00 (1.67, 5.00)	6.67 (6.67, 6.67)	0.31
Seizure	1.70 (1.70, 3.30)	3.30 (1.70, 14.30)	0.22
Weight Loss	5.00 (3.30, 6.70)	10.30 (6.30, 14.30)	0.67
Other	1.70 (1.70, 5.00)	2.80 (2.20, 11.80)	0.08
Neonates			
Common cold	3.33 (1.67, 5.00)	2.78 (2.22, 9.29)	0.44
Cough	5.00 (3.30, 6.70)	8.30 (5.00, 10.00)	0.004
Difficulty breathing	3.00 (2.00, 5.00)	4.00 (2.00, 14.00)	0.01
Fever	5.00 (3.30, 6.70)	8.60 (6.30, 20.40)	<0.001
Refusal to eat, drink, or breastfeed	5.00 (3.30, 6.70)	10.00 (4.40, 21.40)	<0.001
Pus draining from ear	1.67 (1.67, 3.33)	14.29 (14.29, 14.29)	0.11
Vomiting	3.33 (1.67, 3.33)	2.50 (1.67, 13.21)	0.78
Abdominal Pain	3.00 (2.00, 5.00)	31.00 (23.00, 41.00)	0.01
Anemia	3.33 (1.67, 5.42)	-- (--, --)	--
Diarrhea	1.70 (1.70, 2.50)	17.80 (17.80, 17.80)	0.13
Fussiness	2.00 (2.00, 2.00)	3.00 (3.00, 11.00)	0.10
Jaundice	5.00 (3.30, 6.70)	40.20 (24.50, 55.80)	0.07
Rash	1.67 (1.67, 2.50)	6.67 (6.67, 6.67)	0.23
Weight Loss	8.33 (8.33, 8.33)	18.33 (18.33, 18.33)	>0.99
Other	2.50 (1.67, 5.00)	4.44 (3.89, 9.37)	0.20
Infants and Children			
Common cold	5.00 (1.67, 5.42)	11.83 (2.08, 30.36)	0.39
Cough	5.00 (3.30, 6.70)	13.30 (5.40, 25.40)	<0.001
Difficulty breathing	3.00 (2.00, 5.00)	3.00 (2.00, 10.00)	0.35
Fever	5.00 (3.30, 6.70)	6.70 (3.30, 30.00)	0.002
Refusal to eat, drink, or breastfeed	5.00 (3.33, 6.67)	6.67 (2.22, 14.58)	0.07
Pus draining from ear	1.67 (1.67, 5.00)	-- (--, --)	--
Vomiting	3.30 (1.70, 5.00)	3.90 (2.50, 11.80)	0.48
Abdominal Pain	3.00 (2.00, 5.00)	16.00 (2.00, 24.00)	0.34
Anemia	5.83 (4.17, 6.67)	10.00 (10.00, 10.00)	0.28
Diarrhea	3.33 (1.67, 5.00)	2.22 (1.94, 8.25)	>0.99
Fussiness	3.33 (3.33, 5.00)	-- (--, --)	--
Injury	3.33 (1.67, 3.33)	-- (--, --)	--
Jaundice	8.30 (5.00, 10.00)	12.40 (7.90, 16.90)	0.80
Rash	5.00 (5.00, 5.80)	-- (--, --)	--
Seizure	1.70 (1.70, 3.30)	3.30 (1.70, 14.30)	0.22
Weight Loss	1.67 (1.67, 1.67)	2.22 (2.22, 2.22)	>0.99
Other	2.00 (2.00, 5.00)	2.00 (2.00, 19.00)	0.17

*Median is days with reported symptom and denominator is days of follow up (i.e., 60 days for those who survived and up until death for those who died).

Kisenge R, *et al. BMJ Paediatrics Open* 2024; 8:e002613. doi: 10.1136/bmjpo-2024-002613

Supplemental Table 3. Comparison of timing of health care seeking among participants who died and those who survived up to 60 days following hospital discharge

Type of Healthcare Sought	Survived 60 Days After Discharge, n (%)	Died within 60 Days After Discharge, n (%)	*P* value
Day 7			
Hospital	78 (2.1%)	21 (16%)	<0.001
Clinic	30 (0.8%)	3 (2.3%)	0.09
Pharmacy/Herbs	741 (20%)	31 (24%)	0.22
None but had symptoms	68 (1.8%)	4 (3.1%)	0.30
None and had no symptoms	2,834 (76%)	72 (56%)	<0.001
Day 14			
Hospital	101 (2.7%)	17 (16%)	<0.001
Clinic	20 (0.5%)	3 (2.8%)	0.03
Pharmacy/Herbs	453 (12%)	26 (24%)	<0.001
None but had symptoms	75 (2.0%)	4 (3.7%)	0.28
None and had no symptoms	3,139 (83%)	60 (55%)	<0.001
Day 30			
Hospital	119 (3.2%)	20 (24%)	<0.001
Clinic	94 (2.5%)	3 (3.6%)	0.47
Pharmacy/Herbs	726 (19%)	13 (15%)	0.39
None but had symptoms	73 (1.9%)	1 (1.2%)	>0.99
None and had no symptoms	2,766 (73%)	47 (56%)	<0.001
Day 45			
Hospital	90 (2.5%)	19 (33%)	<0.001
Clinic	27 (0.7%)	1 (1.8%)	0.35
Pharmacy/Herbs	425 (12%)	7 (12%)	0.90
None but had symptoms	83 (2.3%)	4 (7.0%)	0.04
None and had no symptoms	3,008 (83%)	27 (47%)	<0.001
Day 60			
Hospital	87 (2.1%)	16 (42%)	<0.001
Clinic	60 (1.4%)	4 (11%)	0.002
Pharmacy/Herbs	230 (5.5%)	2 (5.3%)	>0.99
None but had symptoms	59 (1.4%)	4 (11%)	0.002
None and had no symptoms	3,748 (90%)	14 (37%)	<0.001

Kisenge R, *et al. BMJ Paediatrics Open* 2024; 8:e002613. doi: 10.1136/bmjpo-2024-002613

Supplemental Table 4. Symptoms present during follow up calls among participants who left the hospital against medical advice

Symptoms Reported by Caregiver	Survived 60 Days After Discharge, n (%)	Died within 60 Days After Discharge, n (%)	*P* value
None	32 (26%)	2 (10%)	0.16
Common cold	27 (22%)	3 (15%)	0.77
Cough	73 (58%)	10 (50%)	0.48
Difficulty breathing	1 (0.8%)	6 (30%)	<0.001
Fever	78 (62%)	13 (65%)	0.82
Refusal to eat, drink, or breastfeed	59 (47%)	16 (80%)	0.006
Pus draining from ear	0 (0%)	0 (0%)	--
Vomiting	3 (2.4%)	2 (10%)	0.14
Abdominal pain	0 (0%)	2 (10%)	0.018
Anemia	0 (0%)	0 (0%)	--
Diarrhea	1 (0.8%)	0 (0%)	>0.99
Fussiness	0 (0%)	0 (0%)	--
Injury	0 (0%)	0 (0%)	--
Jaundice	0 (0%)	0 (0%)	--
Rash	2 (1.6%)	0 (0%)	>0.99
Seizure	0 (0%)	0 (0%)	--
Weight loss	0 (0%)	1 (5.0%)	0.14
Other	0 (0%)	0 (0%)	--

Kisenge R, *et al. BMJ Paediatrics Open* 2024; 8:e002613. doi: 10.1136/bmjpo-2024-002613
